# Increased lymphocyte activation and atherosclerosis in CD47-deficient mice

**DOI:** 10.1038/s41598-019-46942-x

**Published:** 2019-07-23

**Authors:** Daniel Engelbertsen, Anu Autio, Robin A. F. Verwilligen, Marie A. C. Depuydt, Gail Newton, Sara Rattik, Erik Levinsohn, Gurpanna Saggu, Petr Jarolim, Huan Wang, Francisco Velazquez, Andrew H. Lichtman, Francis W. Luscinskas

**Affiliations:** 1 Center for Excellence in Vascular Biology, Department of Pathology, Brigham and Women’s Hospital and Harvard Medical School, Boston, MA 02115 USA; 2 Department of Clinical Sciences, Skåne University Hospital, Lund University, SE-205 02 Malmö, Sweden

**Keywords:** Chronic inflammation, Atherosclerosis

## Abstract

CD47, also known as integrin-associated protein (IAP), is a transmembrane protein with multiple biological functions including regulation of efferocytosis and leukocyte trafficking. In this study we investigated the effect of CD47-deficiency on atherosclerosis using a model of adeno-associated virus (AAV)-induced hypercholesterolemia. We observed increased plaque formation in CD47 null mice compared to wild-type controls. Loss of CD47 caused activation of dendritic cells, T cells and natural killer (NK) cells, indicating an important role for CD47 in regulating immunity. In particular, *Cd47* deficiency increased the proportion of IFN-γ producing CD90^+^ NK cells. Treatment with depleting anti-NK1.1 monoclonal antibody (mAb), but not depleting anti-CD4/CD8 mAbs, equalized atherosclerotic burden, suggesting NK cells were involved in the enhanced disease in *Cd47* deficient mice. Additional studies revealed that levels of CD90^+^ and IFN-γ^+^ NK cells were expanded in atherosclerotic aorta and that CD90^+^ NK cells produce more IFN-γ than CD90^-^ NK cells. Finally, we demonstrate that anti-CD47 (MIAP410) causes splenomegaly and activation of DCs and T cells, without affecting NK cell activation. In summary, we demonstrate that loss of CD47 causes increased lymphocyte activation that results in increased atherosclerosis.

## Introduction

CD47 (also known as integrin associated protein, IAP) is a transmembrane protein with diverse functions: interacting *in cis* with integrins to promote integrin-ligand binding and *in trans* with signal regulatory protein-α (SIRPα) and thrombospondin^1^. The interaction between CD47 and SIRPα provides a ‘don’t-eat-me’ signal, the lack of which directs circulating cells for clearance by splenic phagocytes^2^. Anti-CD47 antibodies that increase phagocytosis of tumor cells are currently being tested in clinical trials of patients with leukemia. However, recent studies have suggested that the anti-tumor effect of anti-CD47 treatment is also dependent on T cell activation^3^ and loss of CD47-SIRPα interaction between red blood cells and DCs triggered activation of both T cells and DCs^4^. Accordingly, *Cd47*^−/−^ CD4^+^ T cells are skewed towards a T helper 1 (Th1) phenotype, and *Cd47*^−/−^ DCs produce increased amounts of IL-12p70^5^. Our previous studies have demonstrated that CD47 associates with αLβ2 integrins and that loss of CD47 results in decreased binding of T cells to ICAM-1 and VCAM-1 that results in reduced recruitment *in vivo* and *in vitro* TNFα-induced models of inflammation^6–8^. Indeed, several studies have demonstrated that CD47-deficiency or CD47-Fc protein treatment limits several inflammatory diseases including colitis^9^, bacterial induced arthritis^10^, experimental autoimmune encephalitis^6,11^ and bacterial pneumonia^12^.

Atherosclerosis is an inflammatory disease of arteries caused by deposition of lipoproteins in the vessel wall inducing non-resolving innate and adaptive immune responses^13^. IFN-γ producing lymphocyte subsets (Th1 cells, CD8^+^ cells and NK cells) have been associated with progression of disease while regulatory T cells are associated with reduced plaque burden^14^. During the preparation of this manuscript the role of CD47 in atherosclerosis was not known. However, a recent report by Kojima and colleagues described an athero-protective effect of anti-CD47 antibody blockade by enhancing efferocytosis in atherosclerotic lesions^15^. Here, we present data demonstrating that CD47 plays a protective role in limiting atherosclerotic lesion development, due to a previously unappreciated role for CD47 in regulation of IFN-γ producing NK cells.

## Materials and Methods

### Mice

All animals used in this study were bred and housed in the pathogen-free facility at the New Research Building (Harvard Medical School, Boston, MA). All vertebrate animal related procedures were approved and were carried out in accordance with the Institutional Animal Care and Use Committee (IACUC). Brigham and Women’s Hospital is accredited by AAALAC and has current PHS Animal Welfare Assurance and its animal care facilities and programs meet the requirements of the Federal Law (89–544; 91–579) and NIH regulations. 8–10-week old female WT (C57BL/6) or *Cd47*^−/−^ (C57BL/6 background) mice were given adeno-associated virus (i.v. injection) encoding a gain-of-function proprotein convertase subtilisin/kexin type 9 (AAV-PCSK9^DY^) that causes LDL receptor degradation and promotes hypercholesterolemia in mice fed a western diet^16^. The plasmid was obtained from Addgene (plasmid #58376) and virus stocks were produced at the Boston Children’s Hospital Viral Core. In a pilot experiment we observed that a single-dose AAV-PCSK9^DY^ (5 × 10^11^ genome copies/injection) was sufficient in generating robust hypercholesterolemia over ten weeks of high-cholesterol diet (1.25% cholesterol; HCD, D12108C, Research Diets Inc.) with 922 ± 139 mg/ml total cholesterol after 10 weeks. For experiments with chow-fed male mice, AAV-PCSK9^DY^ was not administered.

### T cell depletion

Female WT or *Cd47*^−/−^ mice were injected i.v. with AAV-PCSK9^DY^ and fed HCD for 10 weeks. After six weeks of HCD, mice were injected i.p. with 100 µg of anti-CD4 (clone GK1.5, BioXCell, West Lebanon, NH, USA) and 100 µg of anti-CD8 mAb (clone 2.43, BioXCell) once a week for 4 weeks before sacrifice.

### NK1.1 depletion

Female WT or *Cd47*^−/−^ mice were injected i.v. with AAV-PCSK9^DY^ and fed HCD for 10 weeks. After 3 weeks of HCD, mice were injected i.p. with 200 µg anti-NK1.1 mAb (clone PK136, BioXcell) twice a week for seven weeks before sacrifice.

### Anti-CD47 treatment

Female WT mice were injected i.v. with AAV-PCSK9^DY^ and fed HCD for eight weeks. After 4 weeks of HCD, mice were given 200 µg anti-CD47 mAb (clone MIAP410, BioXcell) every other day for four weeks until sacrifice. In a short-term study, WT mice or *Cd47*^−/−^ were injected i.v. with AAV-PCSK9^DY^ and fed a HCD for seven weeks, and given 200 µg anti-CD47 mAb every other day for the last 10 days.

### T cell adhesion to immobilized adhesion molecule ligands under shear flow conditions *in vitro*

T-cell adhesion to immobilized recombinant extracellular domain of murine ICAM-1-Fc and SIRPα-Fc (R&D Systems, Minneapolis, MN) in a parallel plate flow chamber was performed as previously described^6,7^. Briefly, 5 × 10^5^/ml T cells in DPBS with 0.1% BSA and 20 mM HEPES were drawn across immobilized ligands under an estimated shear stress of 0.75 dynes/cm^2^ at 37 °C. For mAb blocking studies, T-cells were preincubated at 37 °C for 30 min with function blocking mAb (each at 20 μg/ml) to CD47 (MIAP410), LFA-1 (M17/4, BioLegend), or isotype control murine IgG (Jackson labs).

### Serum lipid analysis

Mouse blood cholesterol was quantified on the c501 module of the Cobas 6000 analyzer (Roche Diagnostics, Indianapolis (IN), USA) using a CHOL2 assay (Roche Diagnostics) to measure total cholesterol levels.

### Tissue processing

Spleens were passed through 70 µm cell strainers (Falcon®, New York (NY), USA) and washed with PBS without Ca^2+^ and Mg^2+^ and treated with ACK lysis buffer. Aortas and livers were cut with micro-scissors and digested for 1 hour at 37 °C with an enzyme mix containing 450 U/mg collagenase I (C0130, Sigma-Aldrich, Saint Louis (MO), USA), 125 U/mg collagenase XI (C7657, Sigma-Aldrich), 60 U/ml hyaluronidase (H3506, Sigma-Aldrich), 60 U/ml DNase1 (D4513-1VL, Sigma Aldrich) with 20 mM HEPES in DMEM (Gibco). Single cells were passed through 70 µm cell strainers, washed with digestion wash containing 0.2% BSA and 2 mM EDTA in PBS without Ca^2+^ and Mg^2+^ followed by a wash with PBS without Ca^2+^ and Mg^2+^. RBCs were lysed using ACK lysis buffer.

### Flow cytometry

Single cell suspensions were analyzed by flow cytometry using the following antibodies: CD3 (17A2), CD4 (RM4-4), CD8 (53–6.7), I-Ab (AF6-120.1), CD44 (IM7), CD62L (MEL14), CD86 (GL-1), CD11b (M1/70), CD11c (N418), CD25 (PC61), NK1.1 (PK136), CD27 (LG.3A10), CD90.2 (53-2.1), Ki67 (16A8), CD127 (A7R34), CD49b (DX5), KLRG1 (2F1/KLRG1) and SIRPα (P84). Zombie Aqua fixable viability dye (Cat. No. 423102, BioLegend, San Diego (CA), USA) was used to exclude all non-viable cells from the analysis. Anti-CD16/CD32 was added to antibody mix to block non-specific mAb binding. FACS buffer containing 0.5% BSA and 0.02% sodium azide in PBS without Ca^2+^ and Mg^2+^ was used for all the washing steps.

To measure intracellular cytokines, splenocytes or aorta digested cells were stimulated with cell activation cocktail containing PMA, ionomycin and BrefeldinA (BrefA) (Cat No. 423303, Biolegend) or given Brefeldin A (Biolegend) alone for 4 hours at 37 °C. Cells were fixed and permeabilized using a fixation and permeabilization kit (Cat. No. 88-8824-00, eBioscience) and subsequently stained with IFN-γ (XMG1.2) or Granzyme B (QA16A02). FACS analysis was performed on a DxP11 flow cytometer (Cytek, Fremont (CA), USA) and the data were analyzed using FlowJo software (FlowJo LLC, Ashland (OR), USA).

### NK cell transfer

WT NK cells were isolated from spleens by magnetic-activated cell sorting (MACS) using the NK cell isolation Kit II (Miltenyi). Sorted cells were labeled with eFluor670 (Thermo-Fisher) per manufacturer’s instructions. Recipient WT or *Cd47*^−/−^ mice were injected i.v. with 5 × 10^5^ isolated NK cells and sacrificed seven days after transfer.

### *In vivo* cytotoxicity assay

Murine lymphoma cell lines RMA (MHC-1 sufficient) and RMA-S (MHC-1 deficient) were labeled using Violet dye (CellTrace™ Violet, Thermo Fisher Scientific), mixed at a 1:1 ratio (5 × 10^5^ cells of each cell type) and i.p. injected into WT and *Cd47*^−/−^ mice (3-5 mice/group). After 48 h the labeled RMA and RMA-S cells were harvested by peritoneal lavage. RMA and RMA-S cells were separated by MHC-I (clone 116505, Biolegend) expression using flow cytometry.

### Cryosectioning

Frozen sections of the aortic root were sectioned using the Leica CM3050S cryostat at 8 µm interval. All images were obtained with a Nikon Microphoto-FXA microscope (Nikon, Tokyo, Japan) equipped with an FX-35-DX- digital camera (Nikon) and ACT-2U imaging software (Nikon). Blinded analysis of the images was performed using image-pro plus software (Media Cybernetics, Rockville (MD), USA).

### Histology

Oil-red-O (Sigma-Aldrich) staining was performed to visualize neutral lipids present in the atherosclerotic lesions. Nuclei were counter-stained using Gill’s Hematoxylin (Sigma-Aldrich). Masson’s trichrome staining was performed using trichrome stain (Masson) kit (HT15-1KT, Sigma-Aldrich) to stain collagen present in the atherosclerotic lesions. Nuclei were stained with Mayer’s Hematoxylin. Necrotic core area was determined by measuring the unstained area within the atherosclerotic plaques stained with Masson’s Trichrome. The percentage of collagen and necrotic core area in the lesions was determined by dividing the collagen or unstained area by the total lesion size. For hematoxylin and eosin (H&E) and Prussian blue staining, spleens were fixed in 10% buffered formalin before sectioning and staining.

### Immunohistochemistry

Macrophage content was stained using MAC-3 antibody as primary antibody (1:900, Cat. No: 553322, purified rat anti-mouse CD107b, BD Pharmingen, San Diego (CA), USA) and biotinylated rabbit anti-rat IgG (1:200, Cat. No. BA-4001, Vector laboratories, Burlingame (CA), USA) as secondary antibody. Sections were incubated with Streptavidin-HRP (Cat. No. K0675, DAKO) and the reaction was visualized using AEC Substrate-Chromogen (Cat. No. K3464, DAKO). Sections were counterstained with Gill’s Hematoxylin to visualize the nuclei. The percentage of macrophage area in the lesions was determined by dividing the macrophage positive area by the total lesion size. To visualize the CD4^+^ T cells present in the atherosclerotic lesion, sections were stained using anti-CD4 as primary antibody (1:90, Cat. No. 550280, purified rat anti-mouse CD4, BD Pharmingen) and biotinylated rabbit anti-rat IgG (1:200, Cat. No. BA-4001, Vector laboratories), USA) as secondary antibody. Sections were incubated with Streptavidin-HRP (Cat. No. K0675, DAKO) and the reaction was visualized using AEC Substrate-Chromogen (Cat. No. K3464, DAKO). To visualize the nuclei, sections were counterstained using Gill’s Hematoxylin. CD4^+^ T cells were quantified by manually counting the stained cells. Terminal deoxynucleotide transferase dUTP nick End labeling (TUNEL) staining was performed to detect apoptotic cells present in the lesion using the APO-BRDU-IHC reagent kit (Cat. No. NBP2-31164, Novus Biologicals, Littleton (CO), USA). The sections were stained per manufacturer’s protocol. Nuclei were visualized using methyl green nuclei staining. TUNEL positive cells were counted manually.

### Statistics

Statistical analysis was performed using Graphpad Prism (GraphPad Software, La Jolla, CA, USA). Significance was calculated using Student’s T-test or non-parametric Mann-Whitney U test when the data was not normally distributed. Probability values < 0.05 are considered as significant.

## Results

### Increased atherosclerosis in *Cd47*^−/−^ mice

**Figure 1:**
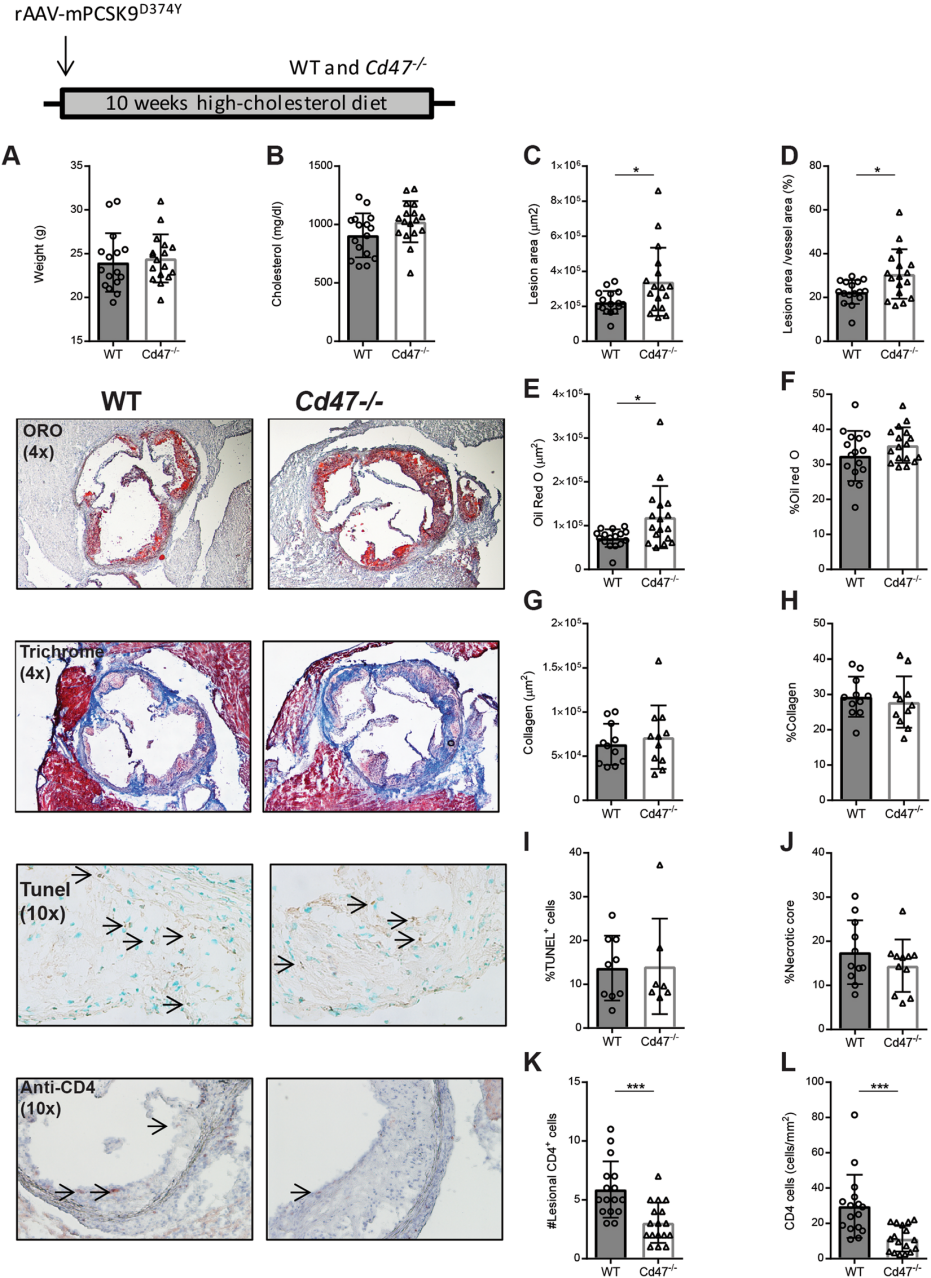
Germline deletion of CD47 increases atherosclerosis, reduces plaque CD4^+^ T cells but does not affect plaque apoptosis. WT and *Cd47*^−/−^ mice (n = 16–17) were injected once with AAV-PCSK9^DY^ and fed high-cholesterol diet for 10 weeks. Weight (**A**) and cholesterol (**B**) were measured at the end of the experiment. Atherosclerosis was assessed in the aortic root. Lesion area (**C**) and lesion area as % of vessel area (**D**). Staining for Oil Red O (**E**,**F**) and Masson’s trichrome staining (**G**,**H**; n = 11). Percentage of lesional TUNEL+ nuclei (**I**; n = 7–9) and plaque necrotic core percentage (**J**). Quantification of lesional CD4^+^ T cells per aortic sinus section (**K**) and density of CD4^+^ T cells normalized by lesion area (**L**; n = 16–17). *p < 0.05, **p < 0.01, ***p < 0.001.

To study the effect of CD47 deficiency on atherosclerosis we injected *Cd47*^−/−^ knockout or wild-type (WT) mice with adeno-associated virus (AAV) containing a gain-of-function mutant form of proprotein convertase subtilisin/kexin type 9 (AAV-PCSK9^DY^)^16^. PCSK9 regulates the levels of LDL receptor (LDLr) on hepatocytes by promoting LDLr degradation and injection of AAV PCSK9^DY^ leads to hypercholesterolemia in mice when fed a high cholesterol diet (HCD)^16^. WT or *Cd47*^−/−^ mice (n = 16–17/group) were injected with a single-dose of AAV-PCSK9^DY^ at 10 weeks of age and fed HCD for 10 weeks before assessment of atherosclerosis (see Supplementary Fig. S1A). At the end of the treatment both groups of mice exhibited similar weight and serum cholesterol concentrations (Fig. 1A,B). We observed increased aortic sinus lesion area (221,941 ± 64,729 vs. 340,309 ± 194,669 µm^2^; p < 0.05) and plaque area to vessel area percentage (22.5 +/− 5.4 vs. 30.7 +/− 11.3%) in *Cd47*^−/−^ mice compared to WT mice (Fig. 1C,D). The proportion of lipid-positive plaque area was not different between groups, while total lipid stained area was increased in *Cd47*^−/−^ mice (Fig. 1E,F). Plaque collagen and macrophage area or levels were not affected by CD47-deficiency (Fig. 1G,H; see Supplementary Fig. S1B,C). We did not observe any differences in the number of TUNEL^+^ nuclei nor a change in necrotic core formation between groups (Figs 1I,J and S1D). In line with the role of CD47 in T cell trafficking, we observed reduced levels of lesional CD4^+^ T cells (Fig. 1K,L) in *Cd47*^−/−^ compared to WT. This finding was further corroborated by flow cytometry of digested aortas showing fewer CD4^+^ and CD8^+^ T cells in *Cd47*^−/−^ compared to WT mice (see Supplementary Fig. S1E-F).

### T cell activation in CD47-deficient mice does not promote atherosclerosis

**Figure 2:**
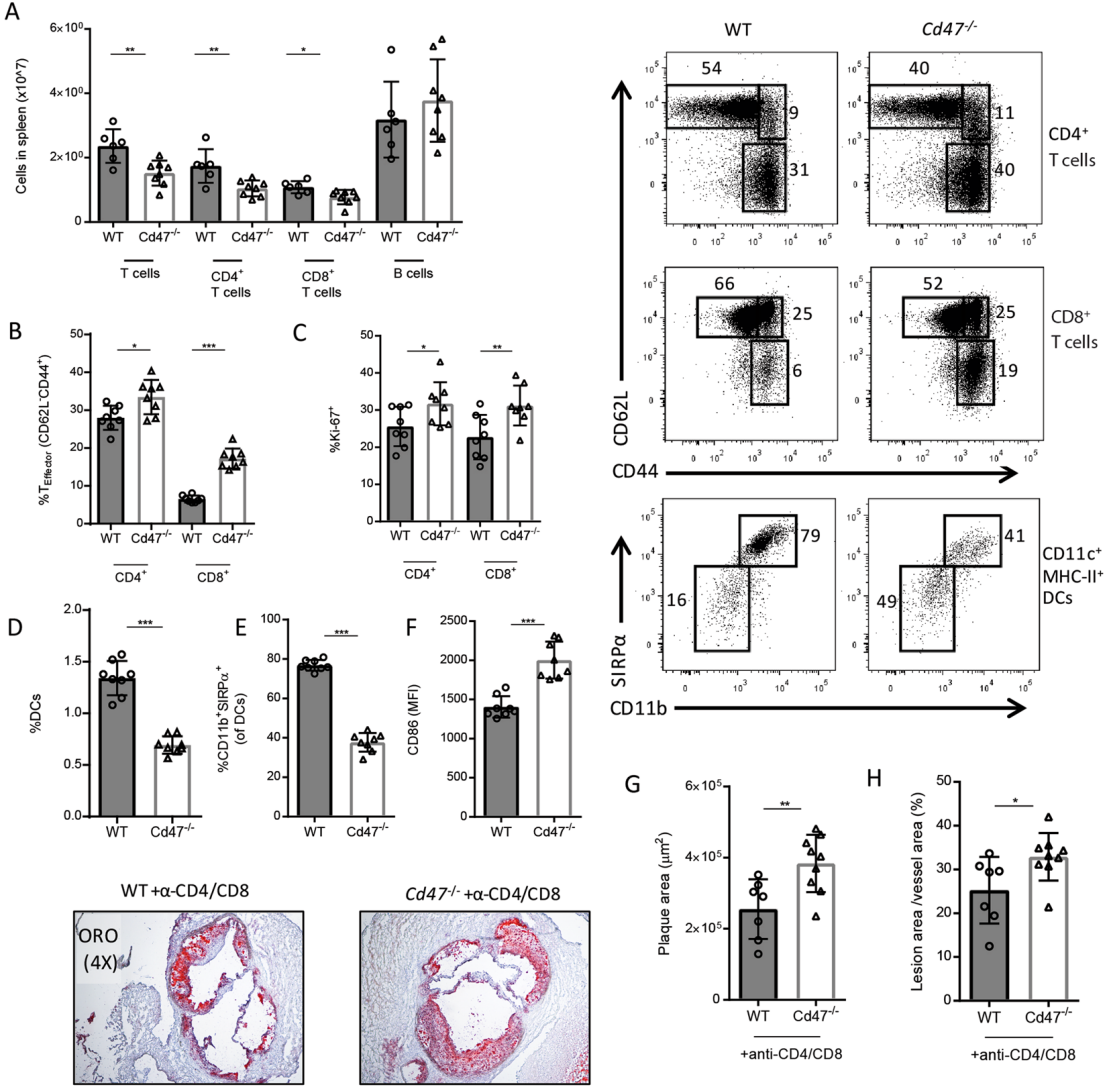
T cell activation does not contribute to aggravated atherosclerosis in *Cd47*^−/−^ mice. WT and *Cd47*^−/−^ mice were injected once with AAV-PCSK9^DY^ and fed high-cholesterol diet for 10 weeks (n = 7–8). Quantification of splenic lymphocytes (**A**). Splenic CD4^+^ and CD8^+^ T effector cells (**B**) and Ki67^+^ cells (**C**). Levels of DCs (**D**), CD11b^+^SIRPα^+^ DCs (**E**) and CD86 mean fluorescence intensity (MFI) on DCs (**F**). Quantification of plaque area and lesion area % of vessel area in WT and *Cd47*^−/−^ mice given depleting anti-CD4/anti-CD8 mAbs (n = 7–9; **G**,**H**). *p < 0.05, **p < 0.01, ***p < 0.001.

Several studies have demonstrated the involvement of the adaptive immunity in atherosclerosis^13,14^. To further investigate the immune phenotype, we performed a follow-up experiment using the same design as above (AAV-PCSK9^DY^ injection and 10 weeks of HCD; n = 8/group). Spleens from *Cd47*^−/−^ mice contained equal number of splenocytes (see Supplementary Fig. S1G). Notably, *Cd47*^−/−^ mice displayed reduced numbers of CD4^+^ and CD8^+^ T cells but similar numbers of B cells (Fig. 2A). *Cd47*^−/−^ mice exhibited increased levels of T effector memory cells (T_EM_ cells; CD62L^-^CD44^hi^) (Fig. 2B) and decreased levels of T naive (T_N_ cells; CD62L^+^CD44^low^) cells consistent with our recent study^6^ (see Supplementary Fig. S1H). Further demonstrating more lymphocyte activation in the *Cd47*^−/−^ mice, levels of proliferating (Ki67^+^) CD4^+^ and CD8^+^ T cells were increased in the spleen (Fig. 2C). Furthermore, in response to phorbol 12-myristate 13-acetate (PMA)/ionomycin/Brefeldin A treatment, more CD8^+^ T cells from *Cd47*^−/−^ mice produced interferon-γ (IFN-γ) than CD8^+^ T cells from WT mice, while percentages of IFN-γ-producing CD4^+^ T cells were not different between groups (see Supplementary Fig. S1I). A similar pattern of T-cell activation in *Cd47*^−/−^ mice was also observed in aorta-draining lymph nodes (see Supplementary Fig. S1J,K). Plasma cytokine and chemokine levels were comparable between groups, the exception was that TNF-α levels were slightly lower in *Cd47*^−/−^ mice as compared to WT control mice (see Supplementary Fig. S1L).

*Cd47*^−/−^ mice have previously been shown to have reduced numbers of dendritic cells (DCs) exhibiting increased levels of activation^4^. Accordingly, we observed a reduction in percentage and total numbers of CD11c^hi^MHC-II^+^ DCs in the spleen of *Cd47*^−/−^ mice (Fig. 2D, see Supplementary Fig. S1M,N). Within the DC compartment, we observed a marked decrease in CD11b^+^SIRPα^+^ conventional DCs from *Cd47*^−/−^ mice and increased surface expression of CD86 (Fig. 2E,F). In a separate cohort of *Cd47*^−/−^mice, we determined that the effects observed were not contingent on hypercholesterolemia because chow fed mice displayed a similar DC and T cell phenotype (see Supplementary Fig. S2A–F). Further, we determined that splenic SIRPα^+^ DCs co-expressed CD4 but were negative for CD8α, while SIRPα^−^ DCs were predominantly CD8α^+^ (see Supplementary Fig. S2G,H), aligning our data with recent reports of loss of CD4^+^ DCs in *Cd47*^−/−^ mice^4^.

Although T cell numbers were reduced in lymphoid tissue and in atherosclerotic lesions, it remained possible that increased activity of remaining T cells could be responsible for the increased atherosclerosis observed in *Cd47*^−/−^ mice. To determine the role of T cells in *Cd47*^−/−^ mice, we depleted CD4^+^ and CD8^+^ T cells in WT or *Cd47*^−/−^ mice using a combination of depleting rat anti-mouse CD4 (GK1.5) and anti-CD8 (2.43) mAb. WT or *Cd47*^−/−^ mice were fed HCD for 10 weeks and treated with CD4/CD8 T cell depletion mAb for the last 4 weeks of the experiment. Depletion of both CD4^+^ and CD8^+^ T cells was efficient in both blood and aorta (see Supplementary Fig. S3A–C). However, T cell depletion did not rescue the phenotype because the *Cd47*^−/−^ mice still displayed increased lesion size and increased plaque area/vessel area percentage as compared to WT mice (Fig. 2G,H).

### Expansion of IFN-γ producing CD90^+^ NK cells in CD47-deficient mice promote atherosclerosis

**Figure 3:**
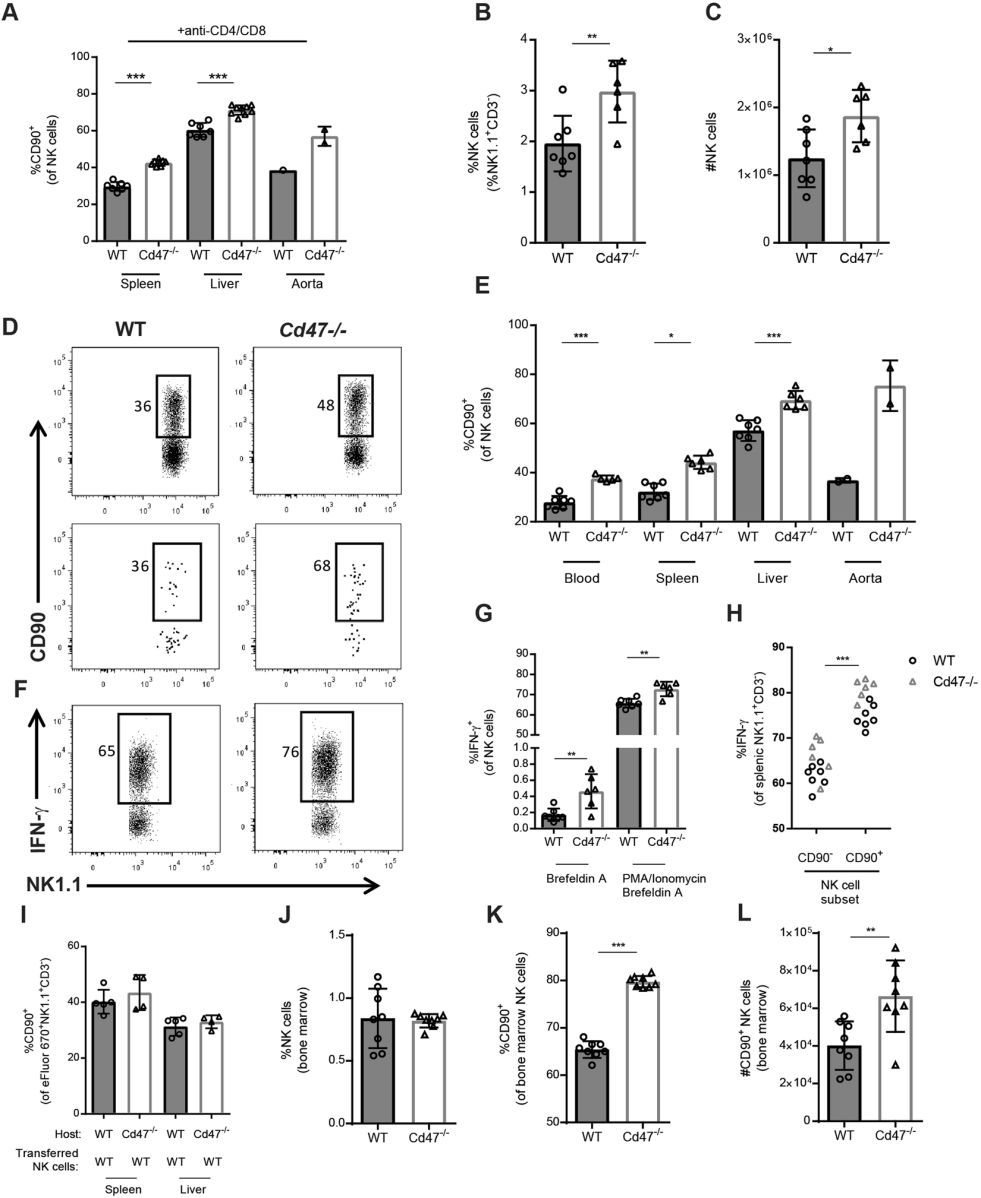
CD47-deficiency mice display increased numbers of CD90^+^ IFN-γ-producing NK cells. Expression of CD90 on NK cells in spleen, liver and aorta of WT and *Cd47*^−/−^ mice given anti-CD4/CD8 depleting mAb (**A**; n = 7–9) WT and *Cd47*^−/−^ mice were injected once with AAV-PCSK9^DY^ and fed a HCD for 8 weeks (n = 7–8). Levels and numbers of splenic NK cells (CD3^−^NK1.1^+^; **B**,**C**). Quantification of CD90^+^ NK cells in blood, spleen, liver and aorta (**D**,**E**). NK cell production of IFN-γ after 4 hours of Brefeldin A or phorbol 12-myristate 13-acetate, ionomycin and Brefeldin A stimulation (**F**,**G**). Comparison of IFN-γ production of CD90^−^ and CD90^+^ NK cells (**H**). WT or *Cd47*^−/−^ mice were injected with eFluor670 labeled WT NK cells. CD90 expression of transferred cells measured seven days after transfer (**I**). Levels of NK cells (**J**), and levels and numbers of CD90^+^ NK cells (**K**,**L**) in bone marrow of untreated WT and *Cd47*^−/−^ mice. *p < 0.05, **p < 0.01, ***p < 0.001.

Given the significant effect of CD47-deficiency on T cell immunity, we examined the impact of CD47-deficiency on subsets of innate lymphocytes. Analysis of spleen and liver leukocytes from CD4/CD8 T cell depleted mice showed a difference in the phenotype of NK cells between *Cd47*^−/−^ and WT mice. Specifically, we observed an increased percentage of CD90^+^ NK cells from spleen and liver, indicating an activated phenotype (Fig. 3A). To expand on and verify this finding, we fed AAV PCSK9^DY^ transduced WT and *Cd47*^−/−^ mice HCD for 8 weeks and analyzed NK cells in lymphoid and non-lymphoid tissue. Percentages and numbers of CD3^−^NK1.1^+^ NK cells were increased in spleens of *Cd47*^−/−^ mice (Fig. 3B,C) while similar NK levels were observed in blood and liver (see Supplementary Fig. S4A). Strikingly, a similar pattern of increased CD90 expression in blood, spleen, liver and aorta was observed (Fig. 3D,E). We note that cell numbers isolated from aortae were low and samples were combined (n = 3–4 aortae per data point) for flow cytometry, which prevented statistical analysis. As a further indication of NK cell activation, the level of CD25 expression was increased (see Supplementary Fig. S4B,C). We found that *Cd47*^−/−^ mice displayed increased levels of CD27^+^ but similar levels of CD127, CD11b and KLRG1 (see Supplementary Fig. S4D). This finding is in agreement with previous reports demonstrating co-expression of CD27 and CD90 on NK cells^17^. NK cells exert their effector function by secretion of cytokines or release of cytotoxic granules for targeted killing of transformed, infected or otherwise stressed cells. CD47-deficency resulted in increased IFN-γ production both at steady state and when stimulated with PMA/ionomycin (Figs 3F,G and S4E). Notably, CD90^+^ NK cells expressed higher levels of IFN-γ compared to CD90^−^ NK cells (Fig. 3H). Levels of Granzyme B^+^ NK cells were not affected by CD47-deficiency (see Supplementary Fig. S4F). The observed NK phenotype, elevated CD90 and increased IFN-γ production, was not dependent on hypercholesterolemia as this was also observed in untreated chow-fed mice (see Supplementary Fig. S4G–J). To further determine NK cytotoxic capacity, WT and *Cd47*^−/−^ mice received i.p. injections of fluorescently labeled murine RMA (MHC-I expressing, NK resistant) and RMA-S (MHC-I deficient, NK sensitive) lymphoma cells. After 48 hours, cells were collected by peritoneal lavage and quantified by flow cytometry. We observed increased percentages of RMA-S in the peritoneal lavage of *Cd47*^−/−^ mice compared to WT mice (see Supplementary Fig. S4K–M), however, total numbers of peritoneal NK cells were also decreased in *Cd47*^−/−^ mice (see Supplementary Fig. S4N).

Next, we asked if *Cd47*^−/−^ mice have NK cell extrinsic effects that promote a phenotypic switch that induces CD90 expression on peripheral NK cells, or whether CD47-deficiency affects development of bone marrow NK cells. We injected 5 × 10^5^ eFluor670-labeled WT splenic NK cells into either *Cd47*^−/−^ or WT recipients and analyzed transferred NK cells in liver and spleen 7 days after transfer. Notably, transferring WT NK cells into a *Cd47*^−/−^ host did not elevate the levels of CD90^+^ NK cells among the transferred NK cells (Fig. 3I). In a separate experiment, we isolated bone marrow from WT or *Cd47*^−/−^ mice. We observed similar levels of total NK cells in bone marrow in both groups, but increased percentage and numbers of bone marrow CD90^+^ NK cells in the *Cd47*^−/−^ mice (Fig. 3J–L). These two experiments suggested that CD47-deficiency skews development of immature NK cells into a CD90^+^ phenotype.

**Figure 4:**
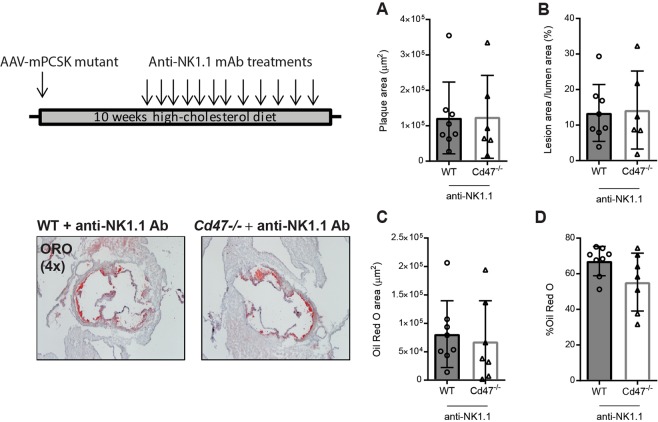
Anti-NK1.1 mediated NK cell depletion abrogates the increased atherosclerosis observed in *Cd47*^−/−^ mice. WT and *Cd47*^−/−^ mice (n = 6–8) were injected once with AAV-PCSK9^DY^ and fed a HCD for 10 weeks and injected with anti-NK1.1 mAb for the last 7 weeks. Aortic sinus lesion area (**A**) and lesion area/vessel area percentage quantification (**B**). Plaque area stained with Oil Red O (**C**) and percentage of Oil Red O stained area (**D**).

To determine whether NK cell activation contributed to the increased lesion burden observed in *Cd47*^−/−^ mice we treated mice with murine anti-NK1.1 mAb (0.2 mg/injection, twice weekly) that depletes NK1.1 expressing cells like NK cells and NK-T cells. Mice were injected with AAV-PCSK9^DY^, fed HCD for 10 weeks and treated with NK1.1 mAb for the last seven weeks of the experiment. NK1.1 treatment rescued the phenotype and generated an outcome of similar lesion size, plaque area/vessel area percentage and Oil Red O levels in WT and *Cd47*^−/−^ mice (Fig. 4A–D).

**Figure 5:**
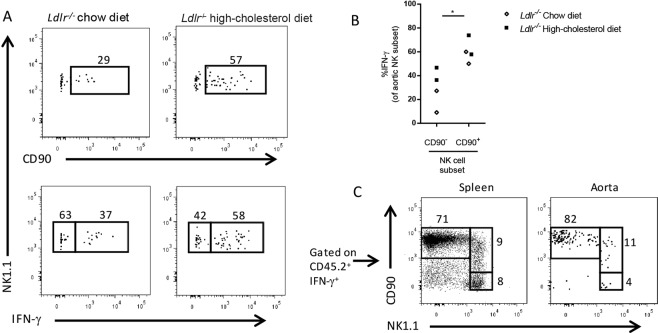
Hypercholesterolemia promotes increased levels of aortic CD90^+^IFN-γ ^+^ NK cells in *Ldlr*^−/−^ mice. (**A**–**C**) *Ldlr*^−/−^ mice were fed chow or high-cholesterol diet for 10 weeks (n = 7–8). Aortas were pooled (n = 3–4 aortas/pool), digested, stimulated with PMA/ionomycin/Brefeldin A and analyzed by flow cytometry for CD90^+^ NK cells and IFN-γ^+^ NK cells (A; representative example of two experiments). Comparison of IFN-γ production by CD90^−^ and CD90^+^ aortic NK cells (**B**). Stimulated aortic cells were gated for CD45.2^+^IFN-γ^+^ and analyzed for CD90 and NK1.1 and showing representative spleen and aorta from high-cholesterol diet fed mice (**C**). *p < 0.05, **p < 0.01, ***p < 0.001.

To broaden our findings, we tested whether CD90 expression is a marker of IFN-γ producing NK cells in aortas of CD47-sufficient host. We fed *Ldlr*^−/−^ mice chow or HCD for 10 weeks and investigated aortic NK CD90-expression and IFN-γ production (for gating, see Supplementary Fig. S5). We observed an increased level of CD90 expression on aortic NK cells in HCD fed mice compared to chow fed mice (n = 3–4 aortae pooled/treatment; Fig. 5A). Like *Cd47*^−/−^ mice, CD90 expression was associated with increased amount of IFN-γ production (Fig. 5B). Further, we determined that CD90^+^NK1.1^+^ cells account for ~10% of IFN-γ^+^ cells in the atherosclerotic plaque (Fig. 5C).

### Anti-CD47 triggers DC and T cell activation but does not affect NK phenotype

During the preparation of this manuscript a study was published that demonstrated that injections of anti-CD47 mAb (clone: MIAP410) resulted in reduced atherosclerosis due to increased efferocytosis of lesional apoptotic cells^15^. However, this report did not describe any extra-aortic effects of anti-CD47 mAb treatment on the immune system. Thus, we sought to investigate if anti-CD47 mAb treatment recapitulated any of the effects on the immune system observed in *Cd47*^−/−^ mice. WT mice were injected with AAV-PCSK9^DY^, fed high cholesterol diet for 8 weeks and given anti-CD47 (MIAP410) or an isotype control IgG1 mAb every other day for the last 4 weeks of the experiment (0.2 mg/injection; see Supplementary Fig. S6A).

**Figure 6:**
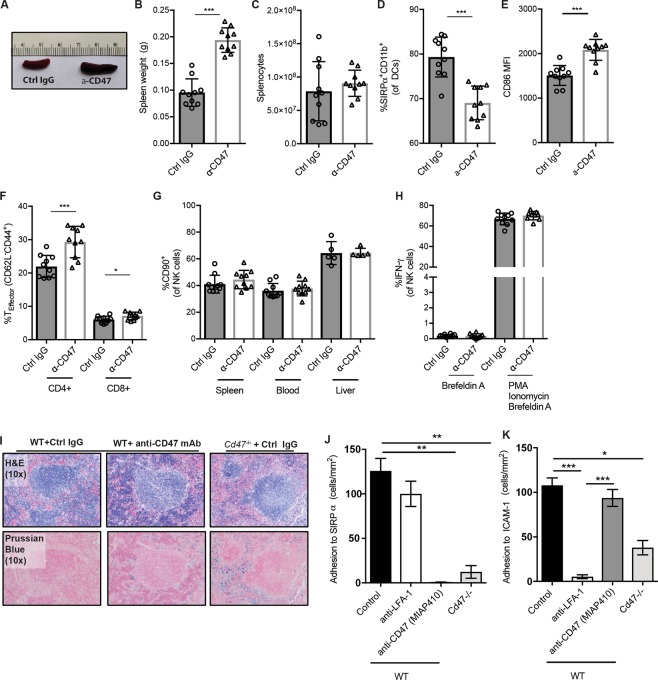
Anti-CD47 triggers DC and T cell activation but does not affect NK phenotype. WT mice were injected once with AAV-PCSK9^DY^ and fed HCD for 10 weeks and treated with anti-CD47 mAb (MIAP410) for the last 4 weeks of the experiment (n = 9–10). Spleens from mice treated with control IgG (ctrl IgG) or anti-CD47 mAb (**A**). Quantification of spleen weight (**B**) and number of splenocytes (**C**). Percentage of SIRPα^+^CD11b^+^ DCs (**D**) and CD86 mean fluorescence intensity (**E**) of DCs. CD4^+^ and CD8^+^ T effector cells (**F**). CD90^+^ expression of NK cells in spleen, blood and liver (**G**). Levels of IFN-γ^+^ NK cells after stimulation with Brefeldin A or PMA/ionomycin/Brefeldin A (**H**). H&E and Prussian blue staining of spleens from WT and *Cd47*^−/−^ mice treated with control IgG or anti-CD47 (**I**). *In vitro* adhesion assay of WT or *Cd47*^−/−^ T cell binding to immobilized ligands SIRPα (**K**) and ICAM-1 (**L**) after pretreatment with control IgG, anti-LFA1 or anti-CD47 mAb (n = 3–5). *p < 0.05, **p < 0.01, ***p < 0.001.

Strikingly, as also described by Kojima *et al*., mice given MIAP410 displayed splenomegaly but normal splenocyte counts (Fig. 6A–C). Like *Cd47*^−/−^ mice, we observed that WT mice treated with anti-CD47 mAb displayed reduced levels of total DCs (see Supplementary Fig. S6B), reduced levels of SIRPα^+^CD11b^+^ DCs (Fig. 6D) and an increased level DC CD86 expression (Fig. 6E). Further, treatment with anti-CD47 increased levels of splenic CD4^+^ and CD8^+^ T_EM_ cells (Fig. 6F). In the spleen, anti-CD47 treatment caused elevation of the percentages of neutrophils and a reduction in B cells and NK cells levels, while in the blood the percentages of CD4^+^ T cells and NK cells were reduced (see Supplementary Fig. S6C–D). However, absolute counts of splenic NK cells were not affected (see Supplementary Fig. S6E). We did not observe any effect of anti-CD47 treatment on NK cell CD90 expression or IFN-γ production (Fig. 6G,H), demonstrating that CD47-blockade in adult mice does not phenocopy the effects of CD47-deficiency on NK cells.

In a separate short-term study (0.2 mg MIAP410 mAb/injection, every other day for 10 days), we analyzed splenic architecture comparing WT mice treated with anti-CD47 or control IgG mAb and *Cd47*^−/−^ mice given control IgG. Histological analysis and Prussian blue staining revealed increased iron deposition in the red pulp area of the spleen of anti-CD47 treated WT mice and *Cd47*^−/−^ mice (Fig. 6I and see Supplementary Fig. S6F), reflecting increased uptake of RBCs by red pulp macrophages after loss of CD47 “don’t eat me” signaling.

CD47 interacts *in cis* with integrins LFA-1 and VLA-4 and *in trans* with receptors on other cells (e.g. SIRPα). Finally, we compared the effect of anti-CD47 blockade (MIAP410) versus CD47-deficiency on lymphocyte binding to immobilized ligands SIRPα and ICAM-1, using an *in vitro* binding assay under flow conditions. Anti-CD47 (MIAP410) ablated interaction between CD47 and SIRPα (Fig. 6J), in line with previous reports^18^, but did not significantly affect T cell binding to immobilized ICAM-1 (Fig. 6K). As has been demonstrated before, *Cd47*^−/−^ T cells did not bind to SIRPα and exhibited a 65% reduction in binding to immobilized ICAM-1^7^.

## Discussion

The main findings are (i) that CD47-deficiency leads to an increase in atherosclerosis, (ii) that CD47-deficiency affects immune cell homeostasis and promotes NK cell CD90-expression and IFN-γ production, and (iii) that treatment with anti-NK1.1 antibodies rescues the increased atherosclerosis observed in *Cd47*^−/−^ mice. Recent studies have highlighted that CD47 may not only regulate macrophage phagocytosis but also both adaptive and innate immune parameters^3,4^. Our study adds NK cells to the growing list immune cells affected by CD47.

Several studies have proposed a pro-atherogenic role of NK cells in atherosclerosis^19^. Transfer of NK cells into lymphocyte-deficient *Apoe*^−/−^*Rag2*^−/−^*Il2rg*^−/−^ resulted in increased lesion formation compared to untreated control mice and depletion of NK cells (and basophils) by anti-Asialo-GM1 antibody treatment resulted in reduced lesion formation^20^. Activating NKG2D ligands are increased in the atherosclerotic lesion and loss of the activating NKG2D receptor lowered serum cholesterol levels and reduced atherosclerosis^21^. Further, it is well established that IFN-γ, has an important role in promoting plaque growth, and IFN-ρ^22^. Treatment of immunodeficient *Rag1*^−/−^*Ldlr*^−/−^ mice with anti-CD90 mAb to deplete innate lymphoid cells resulted in no net effect on atherosclerosis^23^. Our results, that implicate a pro-atherogenic role for CD90^+^ NK cells, suggests that anti-CD90 mAb mediated depletion of CD90^+^ NK cells equalized any harmful effects caused by depletion of atheroprotective ILC2s in the same mice^24^. In the present study, treatment with anti-NK1.1 mAb rescued the increased atherosclerosis in *Cd47*^−/−^ mice, indicating an important role for NK cells in promoting atherosclerosis in mice lacking CD47, although we cannot exclude effects on IL7Rα-dependent type 1 ILCs or NK-T cells that also express NK1.1.

Loss of *Cd47* led to an enrichment of CD90^+^CD27^+^ IFN-γ producing NK cells and depletion of NK cells blocked the increased atherosclerosis observed in *Cd47*^−/−^ mice. Several other groups have previously linked CD27^+^ and CD90^+^ NK cells with increased IFN-γ production^17,25^ that protect against viral infections. We demonstrate that hypercholesterolemia increases the levels of CD90^+^ and IFN-γ producing NK cells in aortas of *Ldlr*^−/−^ mice and that these cells represent ~10% of leukocytes producing IFN-γ after stimulation with phorbol ester and ionomycin. The known inducers of IFN-γ expression by NK cells include IL-12, IL-15, and IL-18 cytokines. These cytokines are produced by multiple cell types, such as macrophages and DCs, found in atherosclerotic lesions^26–29^. Although we did not observe a difference in Granzyme B production by stimulated NK cells comparing groups, we cannot rule out increased NK cytotoxicity, or increased target cell susceptibility, as a contributing factor promoting atherosclerosis in *Cd47*^−/−^ mice. Adoptive transfer of splenic WT NK cells into a CD47-deficient host did not alter the phenotype of the transferred cells suggesting that CD47-deficiency affects earlier NK development. Further study of the potential cell-intrinsic role of CD47 are hampered by the fact that CD47-deficient cells are rapidly phagocytized by splenic macrophages and DCs when transferred into WT hosts^30^. Moreover, although we found elevated levels of IFN-γ producing CD90^+^ NK cells in the aortas of CD47-deficient mice, we cannot exclude the possibility that NK cell atherogenicity in *Cd47*^−/−^ mice is IFN-γ independent and dependent on other mechanisms including cytotoxicity. To test whether cytotoxicity was affected by CD47-deficiency we performed an *in vivo* cytotoxicity assay using clearance of NK-sensitive RMA-S lymphoma as a readout. The results showed diminished clearance of RMA-S cells in *Cd47*^−/−^ mice, indicating a reduced cytotoxic capacity of CD47-deficient NK cells. Since we also observed reduced levels of NK cells in the peritoneum, we cannot exclude that the effect is at least in part due to the reduced abundance of NK cells. Altogether this experiment strengthens the notion that the mechanism by which CD47-deficient NK cells promote development of atherosclerosis is by cytokine production and not through excessive cytotoxic ability.

Previous studies have suggested both pro-atherogenic and anti-atherogenic effects of CD47 and its ligands. Apolipoprotein E (ApoE)-deficient thrombospondin-1 knockout mice displayed increased atherosclerosis compared to *Apoe*^−/−^ control mice, which the authors attributed to increased metalloproteinase activity and defective efferocytosis^31^. Blockade of CD47 (clone MIAP410) in *Apoe*^−/−^ mice fed HCD and given angiotensin II was effective in reducing atherosclerosis and necrotic core formation, however, the atheroprotective effect of anti-CD47 mAb was only modest in a standard model of atherosclerosis akin to that used in our study. There are several possible explanations for the opposing effect on atherosclerosis by anti-CD47 blockade^15^ and CD47-deficiency. First, antibody blockade of CD47 was shown to increase efferocytosis in the lesions by blocking the “don’t eat me” signal that CD47 provides^15^. Recent studies have revealed that CD47-SIRPα is not the only checkpoint regulating phagocytosis of damaged cells and that pro-inflammatory cytokines (IL-17, IL-1β and TNF-α) promote phagocytosis while anti-inflammatory cytokines like IL-10 reduce phagocytic activity against self-cells^32^. Since angiotensin II promotes release of pro-inflammatory cytokines^33^, it is possible that blockade of CD47 in the setting of angiotensin treated hypercholesteremic mice might have a larger impact on efferocytosis and a beneficial effect on atherosclerosis. We anticipated that loss of CD47 would result in decreased necrotic core area or a reduction in apoptotic cells in the plaque, but we did not observe an effect on either of these parameters. Furthermore, if efferocytosis was augmented in *Cd47*^−/−^ mice, the expected outcome would be a reduction in atherosclerosis rather than an increase, as efferocytosis has repeatedly been shown to reduce atherosclerotic lesion formation^34^. This suggests that the atherosclerosis phenotype observed in *Cd47*^−/−^ mice is due to immune activation. Moreover, our results demonstrate that anti-CD47 treatment affects splenic DCs and T cells in a similar manner as seen in *Cd47*^−/−^ mice. Blockade by anti-CD47 mAb has been shown to reduce tumor burden by effects linked to increased phagocytosis of tumor cells^35^, although a recent study has shown that anti-tumor effects of anti-CD47 mAb are dependent on CD8 T cells and DC activation^3^. Importantly, anti-CD47 mAb treatment did not mimic the effect of CD47-deficiency on NK phenotype, which is in line with anti-CD47 mAb treatment and CD47-deficiency having divergent effects on atherosclerosis.

Several caveats to the present study are warranted. Multiple leukocyte subsets are affected in the *Cd47*^−/−^ mouse: SIRPα^+^ DC and T cell numbers are reduced, and the remaining DCs and T cells display an elevated state of activation. A recent study reported that recognition of red blood cell CD47 by SIRPα provides an inhibitory signal that maintains DC phenotype in a quiescent state in WT mice^4^. The authors suggested loss of inhibitory SIRPα signaling in *Cd47*^−/−^ mice is the cause of DC activation. The cause of the T cell phenotype observed in *Cd47*^−/−^ mice is unclear but may be caused by DC activation, or increased rate of apoptosis of activated effector T cells^6^, or due to cell-intrinsic effects of CD47. We tested whether activation T cells explained the increased atherosclerosis in *Cd47*^−/−^ mice by antibody depletion of CD4^+^ and CD8^+^ T cells. *Cd47*^−/−^ mice depleted of T cells still displayed increased lesion size compared to WT mice. Although we cannot rule out a role for T cell subsets, such as regulatory T-cells, in shaping early plaque events, we can conclude that they do not play a crucial role in enhanced plaque formation in *Cd47*^−/−^ mice. Further, since we anticipate that the major function of activated DCs is activation of T cells, but T cell depletion did not abrogate increased lesion size, we propose that DC activation is not a cause for the increased atherosclerosis observed in *Cd47*^−/−^ mice.

In summary, we demonstrate an unexpected protective role for CD47 in murine atherosclerosis. Our data suggest that the primary effect of CD47-deficiency on atherosclerosis is not related to efferocytosis in the plaque but rather due to dysregulation and activation of NK cells.

## Supplementary information

Supplemental Data

## Data Availability

The datasets generated during and/or analyzed during the current study are available from the corresponding author on reasonable request.
