# Supplementary material for: Increased lymphocyte activation and atherosclerosis in CD47-deficient mice
Source: Sci Rep. 2019 Jul 23;9:10608. doi: 10.1038/s41598-019-46942-x (PMC6650435; doi:10.1038/s41598-019-46942-x)
Supplement: Supplementary file 1 — Supplemental Data [file 41598_2019_46942_MOESM1_ESM.pdf]

Increased lymphocyte activation and atherosclerosis in CD47-deficient mice

Daniel Engelbertsen*,†, Anu Autio*, Robin A.F. Verwilligen*, Marie A.C. Depuydt*, Gail Newton*, Sara Rattik†, Erik Levinsohn*, Gurpanna Saggu*, Petr Jarolim*, Huan Wang*, Francisco Velazquez*, Andrew H. Lichtman*,‡, Francis W. Luscinskas*‡

*Center for Excellence in Vascular Biology, Department of Pathology, Brigham and Women's Hospital and Harvard Medical School, Boston, MA 02115 USA; † Department of Clinical Sciences, Malmö University Hospital, Lund University, SE-205 02 Malmö.

‡Joint senior authorship

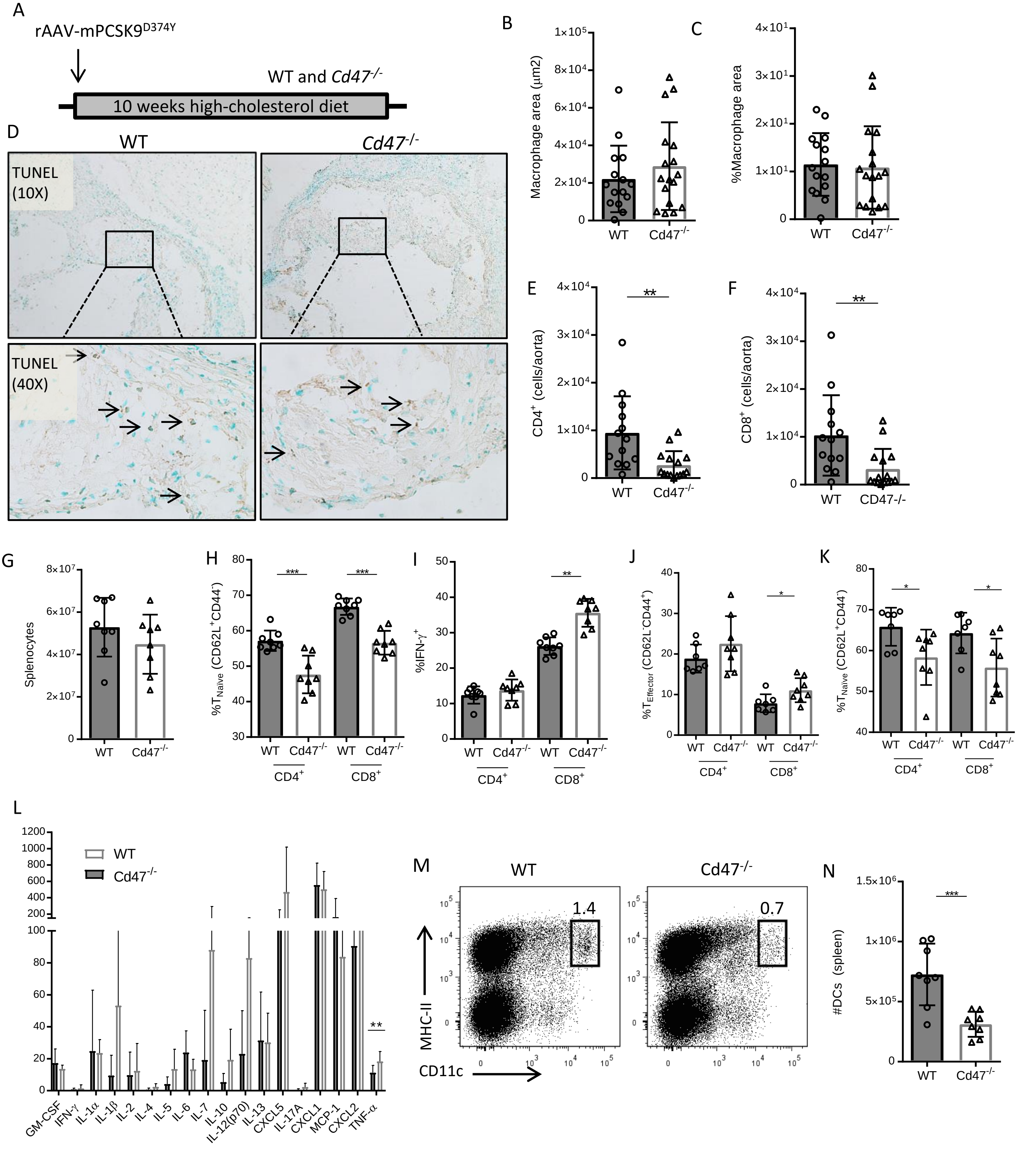

Supplemental Figure 1. Plaque and immune phenotype in Cd47$^{-/-}$ mice

Experimental outline (A). Aortic sinus macrophage area (B) and percent macrophage area (C) as determined by immunohistochemistry. TUNEL staining at either 10x or 40x magnification (D). Aortic CD4^{+} (E) and CD8^{+} (F) T cells determined by flow cytometry. Splenocyte count (G). Percentage of naïve T cells (H) and IFN-γ production of CD4^{+} and CD8^{+} T cells (I). Levels of effector (J) and naïve (K) T cells in aorta-draining LN. Plasma cytokine and chemokine analysis (L; n=6-7 mice/group). Gating example for MHC-II^{+}CD11c^{hi} dendritic cells (M) and splenic DC quantification (N). * $p<0.05$, ** $p<0.01$, *** $p<0.001$

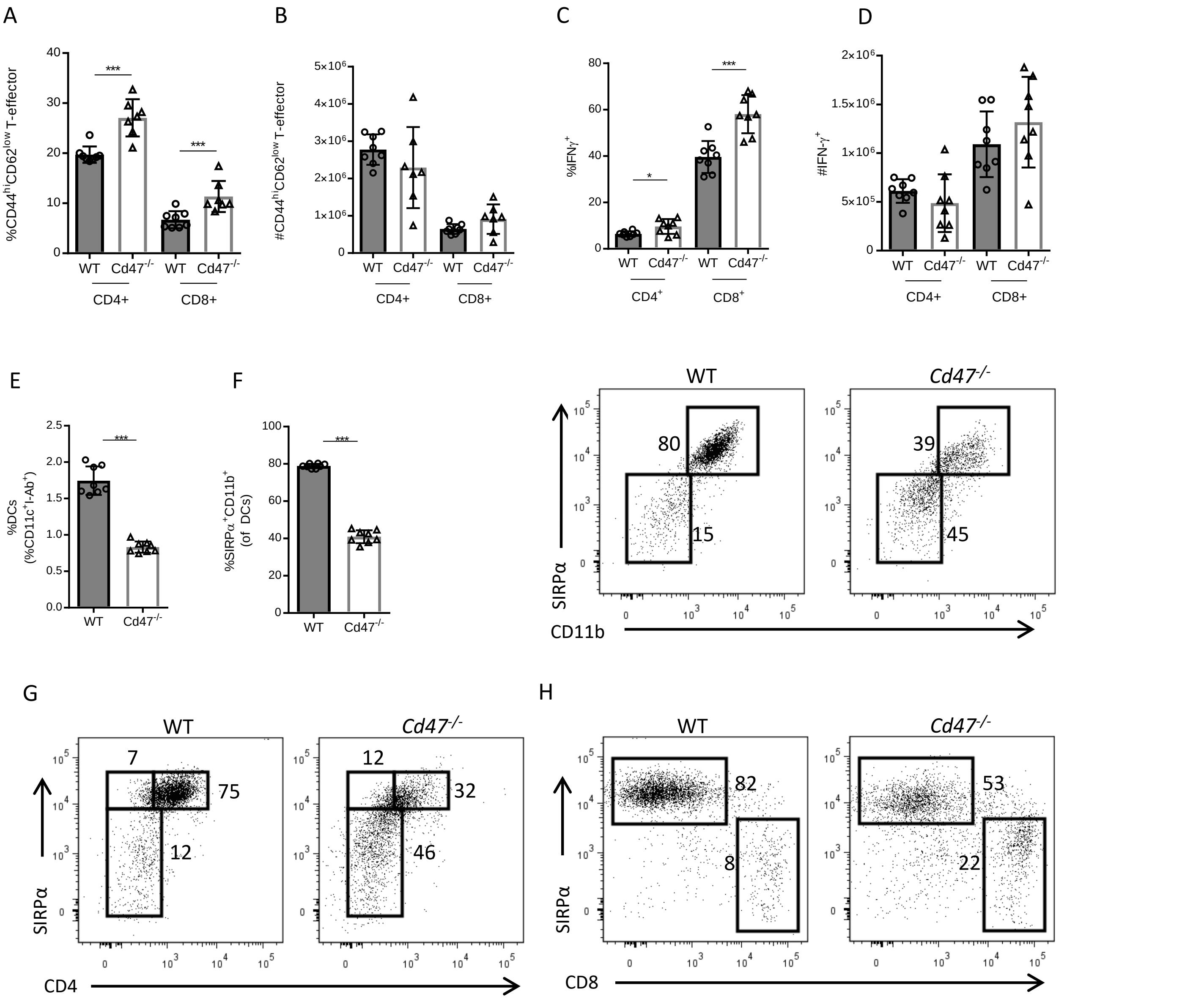

Supplemental Figure 2. Immune perturbations observed in Cd47$^{-/-}$ mice are not contingent on hypercholesterolemia

Splenocytes from chow-fed *Cd47$^{-/-}$* and WT mice were analyzed by flow cytometry. Percentage (A) and numbers (B) of CD44^{hi}CD62L^{low} CD4^{+} and CD8^{+} T-effector cells. Percentage (C) and numbers (D) of IFN-γ-producing CD4^{+} and CD8^{+} T cells. Percentage of DCs (E; MHC-II^{+}CD11c^{hi}) and SIRPα^{+}CD11b^{+} DCs (F) of total DCs. DCs were analyzed for expression of SIRPα vs. CD4 (G) and SIRPα vs. CD8 (H). * $p<0.05$, ** $p<0.01$, *** $p<0.001$

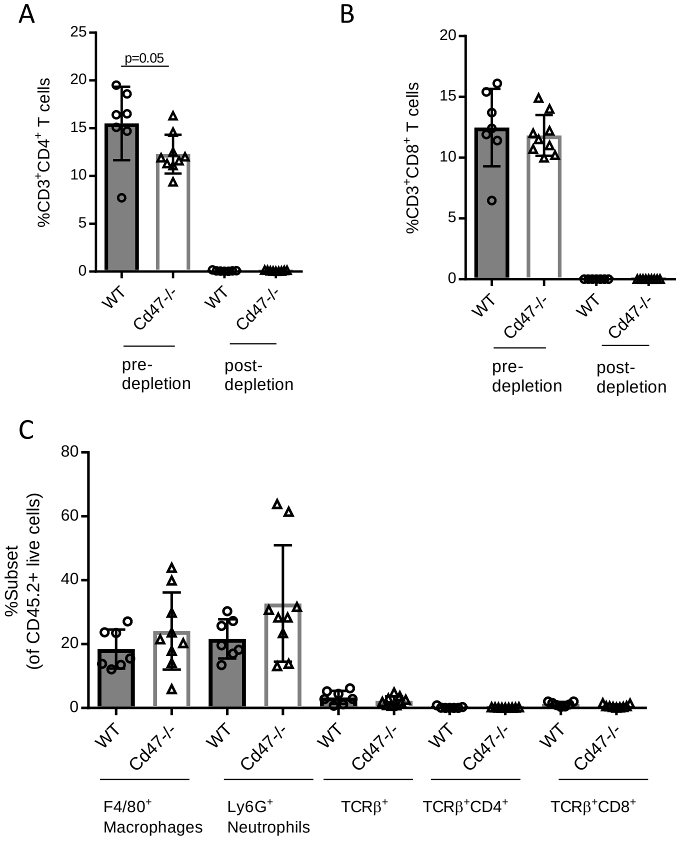

Supplemental Figure 3. Depletion of T-cells after anti-CD4/anti-CD8 treatment

WT and *Cd47*$^{-/-}$ mice (n=7-9) injected with depleting anti-CD4/anti-CD8 mAb were analyzed for T-cells in blood (A-B) and digested aorta (C).

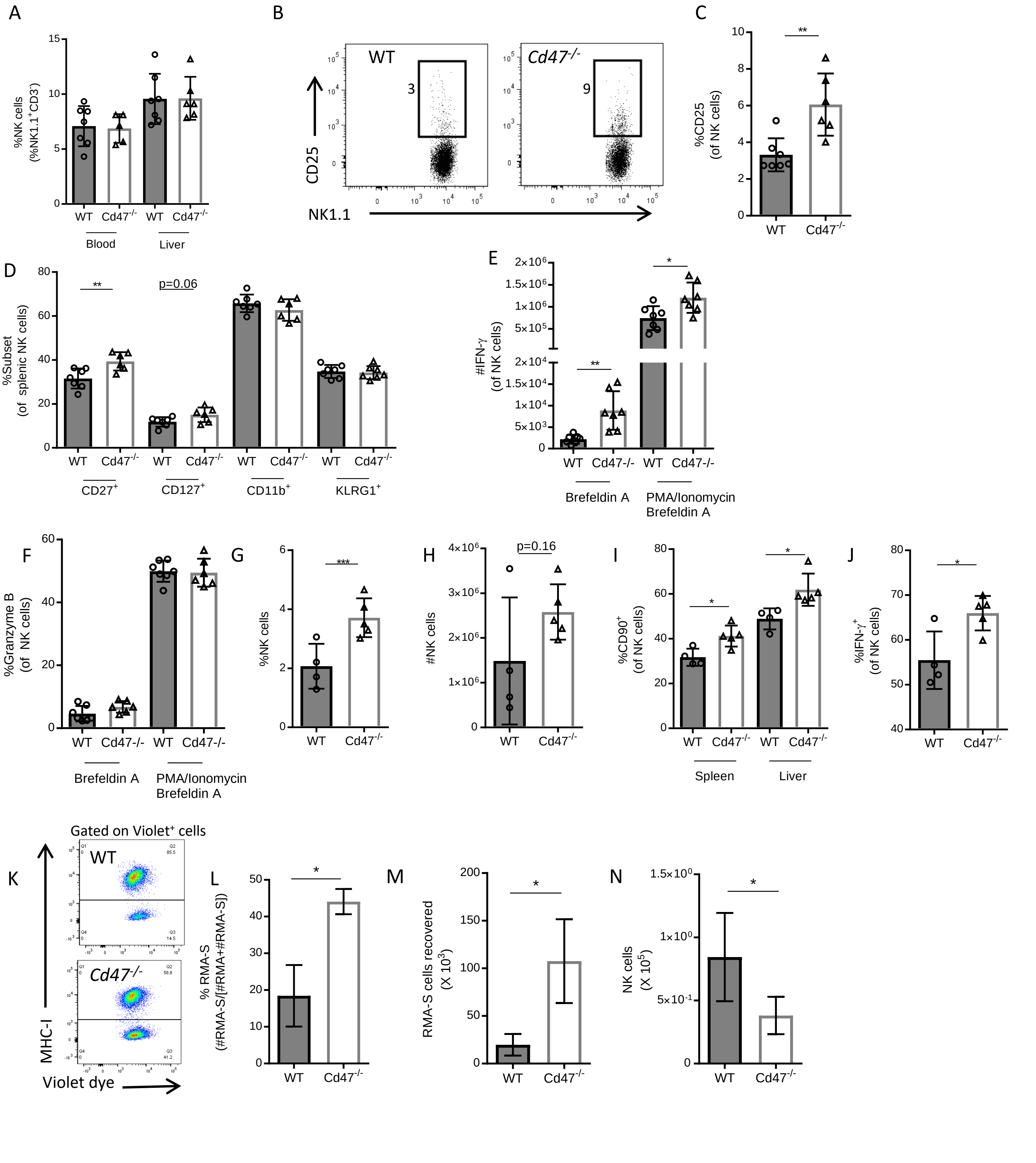

Supplemental Figure 4. CD47-deficiency affects NK phenotype

Cd47$^{-/-}$ and WT mice (n=7-8) were fed high-cholesterol diet for 8 weeks. Percentage of NK cells in blood and liver (A). Gating (B) and quantification of CD25^{+} NK cells in spleen (C). Analysis of CD27, CD127, CD11b and KLRG1 expression on splenic NK cells (D). Numbers of splenic IFNy^{+} NK cells (E) and levels of Granzyme B^{+} NK cells (F) after stimulation with with Brefeldin A or PMA/ionomycin/Brefeldin A. *Cd47$^{-/-}$* and WT mice (n=7-8) were fed chow diet and spleen and liver analyzed for NK cell subsets using flow cytometry (G-J) . Violet labeled RMA and RMA-S cells (1:1 ratio) were co-injected i.p. into *Cd47$^{-/-}$* and WT mice (n=3-5) and recovered 48h later by peritoneal lavage (K). Quantification of %RMA-S (L) #RMA-S (M) and %NK1.1 (N) in peritoneal lavage.
* $p<0.05$, ** $p<0.01$, *** $p<0.001$

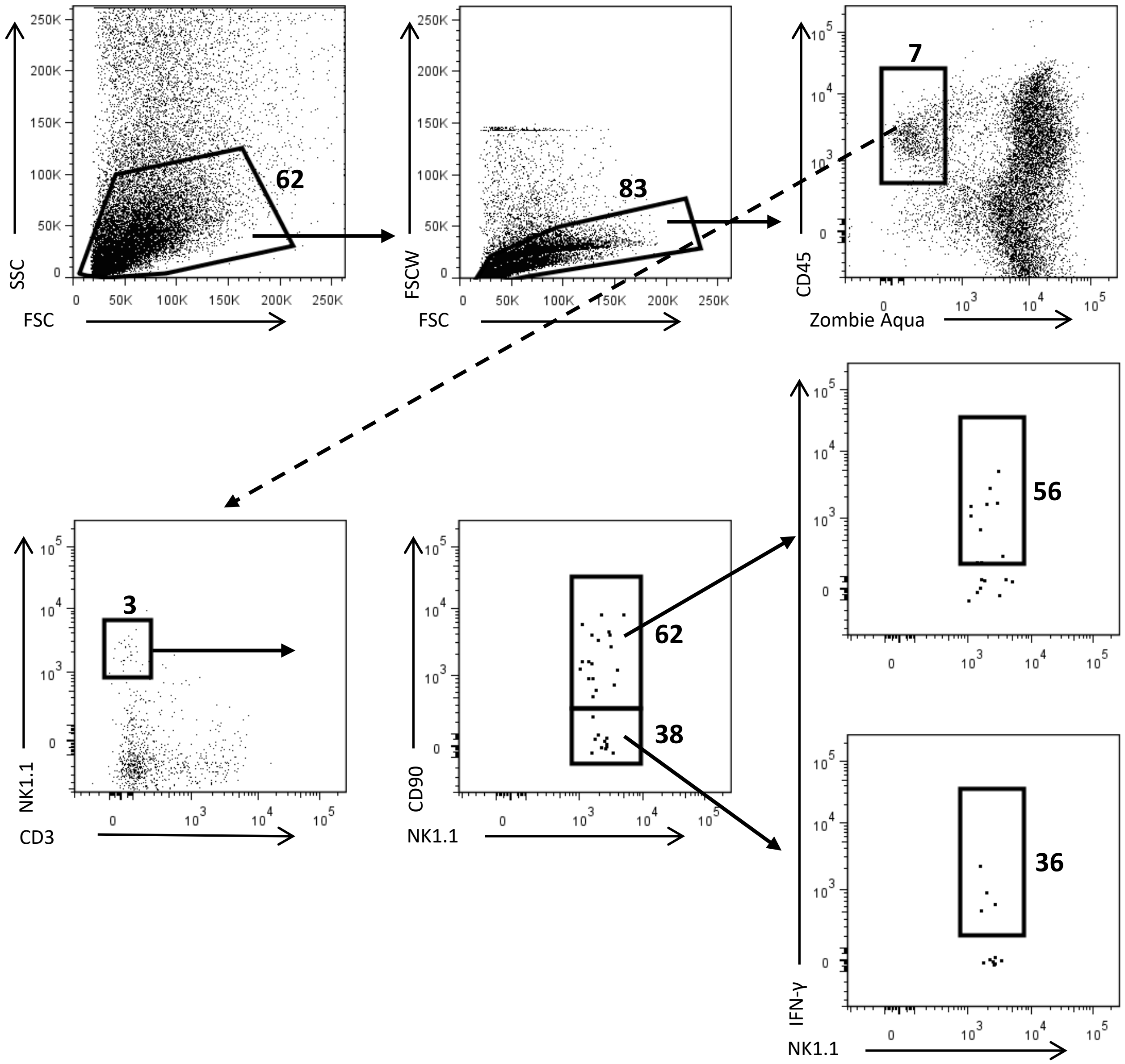

Supplemental Figure 5. Flow cytometry analysis of murine aorta

Aortas from LDLr-/- mice were digested, treated with PMA/ionomycin/Brefeldin A for 4 hours and stained for expression of surface markers and intracellular IFN-γ. Gating for IFN-γ production in $CD90^{+}$ and $CD90^{-}$ NK cells ($CD45^{+}Zombie^{-}NK1.1^{+}CD3^{-}$) shown for .

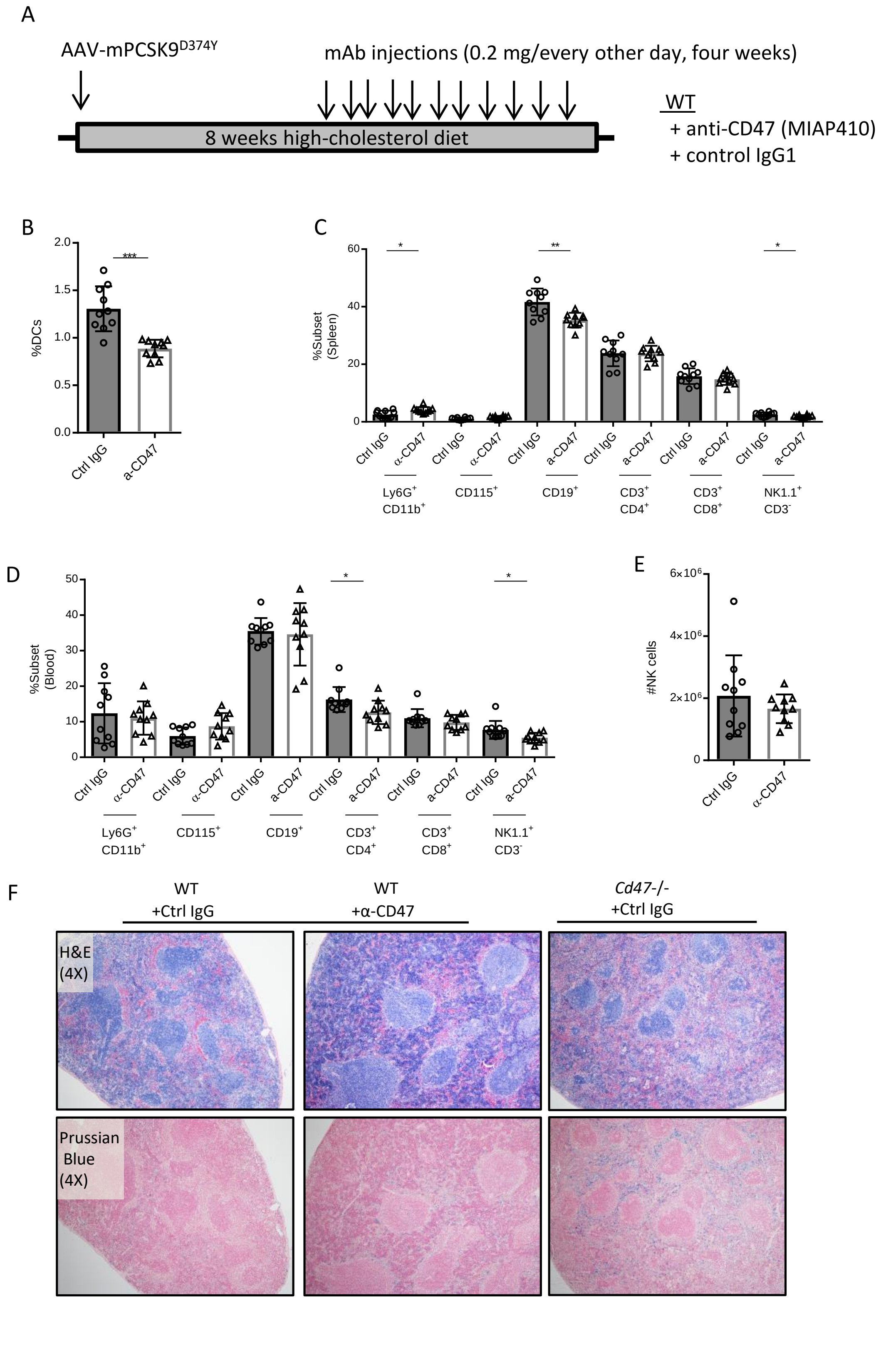

Supplemental Figure 6. Immune phenotype of WT mice treated with anti-CD47 (MIAP410)

Experimental design (A) of anti-CD47 treatment of WT mice given AAV-PCSK9^{DY} and fed high-fat diet (n=10/group). Percentage of splenic DCs (B). Levels of leukocyte subsets in spleen (C) and blood (D). Numbers of NK cells in spleen (E). * $p<0.05$, ** $p<0.01$, *** $p<0.001$
